# Safety Integrity Level (SIL) assessment process for WSN using multi-QoS metrics

**DOI:** 10.1016/j.mex.2025.103660

**Published:** 2025-10-08

**Authors:** Sivasubramanian Srinivasan, T.K. Ramesh, Roberto Paccapeli

**Affiliations:** a Department of Electronics and Communication Engineering, Amrita School of Engineering, Bengaluru, Amrita Vishwa Vidyapeetham India; b Functional Safety Manager in Red Hat, Italy

**Keywords:** Industrial functional safety, QoS metrics, Safety Integrity Level, Wireless Sensor Networks, IoT

## Abstract

Wireless Sensor Networks are inseparable part of Industrial IoT and are deployed throughout the industrial value chain from material procurement to inventory management, storage, process monitoring and control, packaging, delivery till commissioning. In the context of industry 4.0, proliferation of WSN makes it both performance as well as safety critical due to the real time data collection, remote monitoring and machine-to-machine communication for collaboration and autonomous decision making. Deployment of WSN for safety applications thus necessitates compliance of critical QoS metrics with safety integrity levels (SIL) as demanded by the application, for example minimal delay in communication, longer network life, higher throughput, successful data detection rate, packet delivery ratio etc., While there are publications available to illustrate the assessment of safety integrity compliance of WSN, the treatments & illustrations therein are found to be limited to a single QoS metrics. However, in practice, the performance characterization of industrial WSN involves multiple QoS metrics, hence there is a need to address safety assessment use cases involving multiple QoS metrics. In this paper we are trying to bridge this gap by illustrating the safety assessment approach involving more than one QoS metrics. The approach illustrated here leverages random data simulation technique for generating QoS metrics such as the delay bound (DB) and false positive detection rate (FPDR) of a typical WSN in an industrial application environment and the statistical techniques used for consolidation of results for the decision making related to compliance with the safety integrity level. This paper is expected to serve functional safety design engineers for real time safety assessment of WSN involving more than one QoS metrics. We conclude this paper by identifying opportunities for future research in the area of safety integrity assessment of industrial WSN systems using QoS metrics.

• Multiple QoS metrics are leveraged to assess compliance of WSN with safety integrity level (SIL) targets specified in industrial functional safety standard IEC 61508
• The safety compliance of WSN with SIL targets is assessed considering significance of variations in all the applicable QoS metrics based on the p-value statistic.
• In the event of non-compliance of QoS metrics with safety targets, data communication defences to be improved to achieve compliance.

## Graphical abstract

**Figure:**
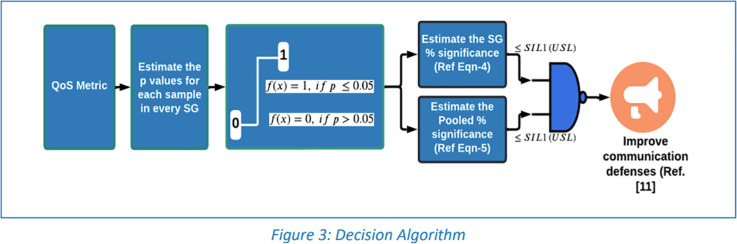

**Specifications table**

**Table:** 

**Subject area**	Engineering
**More specific subject area**	Wireless Sensor Network
**Name of your method**	Safety Integrity Level (SIL) Assessment process for WSN using multi-QoS metrics.
**Name and reference of original method**	S. T. K. R. R. P. a. L. F. Srinivasan, "Industrial functional safety assessment for WSN using QoS metrics," Heliyon, vol. 8, no. 11, p. 9, 2022 S. Srinivasan, T. K. Ramesh, R. Paccapeli and L. Fanucci, "Functional Safety Integrity Assessment for Industrial Wireless Sensor Network Using QoS Metrics," in 2021 IEEE 3rd PhD Colloquium on Ethically Driven Innovation and Technology for Society (PhD EDITS), Bangalore, India, 2021.
**Resource availability**	None

## Background

Wireless Sensor Network (WSN) has become an essential part of industrial IoT serving integration of the product value chain right from material procurement to delivery and successful commissioning or use by customers. Due to the vulnerability of WSN to noise interference, security attacks can lead to data loss, delay, masquerade, low throughput etc., Hence conservatively, in safety applications wireless communication is in general recommended to be used as a backup option. In our viewpoint, WSN systems can very well be deployed for safety applications in industrial systems after assessing the compliance with Safety Integrity Level (SIL) per industrial functional safety requirements. In this paper we are bringing out an approach to carry out safety assessment of a real-time end-to-end industrial WSN where multiple QoS metrics determine the health of the system. QoS metrics are influenced by the hardware characteristics and data processing algorithms of transmitters, nodes, base stations and the noise characteristics of the communication channel or external security attacks. Hence the real time statistical variations in QoS metrics need to be considered to assess safety compliance. Given a scenario of multiple QoS metrics to determine the safety compliance of WSN, the algorithm deployed to determine the special cause induced variations in QoS metrics, and the approach adopted to consolidate variations in multiple QoS metrics become critical to assess compliance with safety integrity goals. A higher confidence in identifying special causes induced significant variation will lead to missing a critical deviation in QoS metrics leading to non-compliance with safety targets and on the other hand, a loosely specified criteria to differentiate a significant variation from variations induced by random factors will result in frequent tuning of the system for safety compliance. In this paper, the safety assessment approach is illustrated in detail by simulating probability distributions of the QoS metrics such as the delay bound (DB) and false positive detection rate (FPDR), the logic used to determine significant vs non-significant variations in QoS metrics, consolidation logic used for the assessment and interpretation of the results for safety compliance assessment. These QoS metrics chosen for illustration are driven by the criticality of these metrics for real time applications in process industries.

In this paper firstly an overview of the Industrial Safety Integrity Level is provided in Section II. In Section-III, the industrial application of QoS metrics is discussed. In Section IV, details of simulation of multi-QoS metrics are provided. In Section-V the safety assessment approach adopted using the results obtained from simulation are brought out and finally, in Section-VI, the conclusions and future opportunities for safety assessment of WSN using QoS metrics are summarized.

## Method details

### Introduction

Wireless Sensor Network (WSN) has become an essential part of industrial IoT serving integration of the product value chain right from material procurement to delivery and successful commissioning or use by customers. Due to the vulnerability of WSN to noise interference, security attacks [1] can lead to data loss, delay, masquerade, low throughput etc., Hence conservatively, in safety applications the wireless communication is used as a backup option [2] and its use is not forbidden [3]. It is brought out that deployment of WSN in industries for the closed loop control and safety applications has brought the value proposition [4]. The survey on an application such as sensor network in [5] mentions key areas of application such as factory process control, industrial automation and manufacturing monitoring. Further, with the industry 4.0 revolution, WSN is an essential element for effective and flexible manufacturing. For example, reliable communication between sensors, actuation systems and computing elements is a key for collaborative robots [6,7]. The deployment of WSN to improve occupational safety and health in underground mines is discussed in [8]. The use case of wireless for safety are related to safety Alerts & Alarms and the applications are gas detection, fire prevention, level detection, safety showers, etc., [9] Given the foregoing application areas of WSN, in our viewpoint WSN systems can very well be deployed for safety applications by carrying out assessment of safety compliance with Safety Integrity Level (SIL) per industrial functional safety requirements [10,11].

As the performance characteristics of WSN in a typical industrial environment is assessed based on multiple QoS metrics, safety compliance assessment needs to consider all the relevant QoS metrics, and the associated statistical variations involved. While there are publications available to illustrate the assessment of safety integrity compliance of WSN, the treatments & illustrations therein are limited to a single QoS metrics. Hence there is a need to illustrate the safety integrity assessment methodology in multi-QoS use case scenarios. In this paper we are trying to bridge this gap by illustrating the safety assessment approach involving more than one QoS metrics. A WSN system encompasses hardware elements such as sensor nodes, processing stations & gateways, destination nodes and actuators. In this paper a process to assess the SIL compliance of WSN based on multiple QoS metrics is brought out. The details on SIL for the hardware systems referred as Electrical/Electronics/Programmable Electronic systems can be referred in [12]. The QoS metrics are influenced by the hardware characteristics and data processing algorithms of transmitters, nodes, base stations and the noise characteristics of the communication channel. Hence the real time statistical variations in QoS metrics need to be considered to assess safety compliance. We believe that the assessment process discussed in this paper can serve the industrial WSN safety design and maintenance engineers to ensure compliance of WSN with SIL.

This paper is organized as follows. First an overview of the Industrial Safety Integrity Level is provided in Section II. In Section-III, the industrial application of QoS metrics is discussed. In Section IV, details of simulation of multi-QoS metrics are provided. In Section-V the safety assessment approach adopted using the simulation results are brought out and finally, in Section-VI, the conclusions and future opportunities for safety assessment of WSN using QoS metrics are summarized.

### Overview on industrial functional safety

Safety is defined as “the condition of being protected from or unlikely to cause danger, risk, or injury.” [13] The safety as applied to the industrial safety sector aim to protect industrial workers, machinery, structures and the environment. Hence, by identifying the hazards and related preventive measures, the potential loss could be minimized. Functional Safety (FS) is part of industrial safety associated with functionality of systems or equipment meant to respond in a way to ensure safety [14]. Functional safety aims to achieve safe operation of the overall system and covers all the functions that are meant to mitigate unsafe system states and prevent an accident from happening that can result in loss of life or loss of property.

The IEC-61508 is the industrial functional safety standard, and it provides guidelines for development of safety systems [15]. The standard differentiates between a safety function (SF) and non-safety functions and defines a metric referred as safety integrity levels (SIL) for safety functions. Meeting the SIL as per IEC-61508 means that the risk associated with a safety system is controlled for safe operations. According to IEC-61508 the faults in E/E/PE systems can be classified as) Random failures and b) Systematic failures. The random failures are associated with hardware elements since the failure rate of components and interface hardware elements are probabilistic in nature. On the other hand, systematic failures depend on the pattern of development, which is nothing but the development process, i.e. method of development. The random causes include but are not limited to excess operating stress, inherently weak parts, human errors, environmental noise etc. The random hardware failures are expressed as the failure rate i.e. number of failures per unit time. With process and technology maturity, products are inherently reliable and have very low failure rates that are of the order of a few failures over a billion operating hours. Hence failure rates are expressed as number of failures per billion operating hours [15,16] in short referred as ‘Failures-In-Time (FIT)’. SIL implies the degree of safety built into the systems performing safety functions. The degree of safety is defined in terms of the failure probability of hardware elements. The highest level of safety implies a lower probability of failure of hardware. The hardware failure probability depends on the operating duration or the operating duty cycle under given operating stress conditions. It may be noted that the operating duty cycle would vary between applications and within a given application. Thus, the safety targets will depend on the type of application and the operating duty cycle. The safety standard [16] provides the guideline on SIL targets considering the demand rate. When the demand rate is higher than one per year, it is considered a high demand system. For example, systems such as pressure regulators, temperature controllers, speed governor etc., are high demand systems since such systems will be under continuous operation. Systems like fire extinguishers, backup servers, over temperature cut off valves etc., are likely to have occasional demands and are classified as low demand systems. The SIL-1 & SIL-2 targets of probability of failure for a high and low demand systems are shown Table 1.

**Table 1:** SIL Targets for low and high demand systems.

Safety Integrity Level	Low Demand	High Demand
	Average Probability of Dangerous Failure on Demand (PFD avg)	Average Frequency of a Dangerous Failure per hour (FPH avg)
SIL 1	10^–2^ ≤ p < 10^–1^	10^–6^ ≤ p < 10^–5^
SIL 2	10^–3^ ≤ p < 10^–2^	10^–7^ ≤ p < 10^–6^

The quantitative requirements for SIL-3 & 4 can further be referred to in section-7 of [15]. The systematic faults are caused by non-adherence to the quality management (QM) principles during the development of safety products. The QM principles include but are not limited to requirements traceability, configuration management, change management, root cause analysis and corrective action system etc., The requirements to mitigate systematic faults can be referred to in Annexure B of [16]. The systematic capability is assessed considering the method of development adopted for safety systems. For example, hardware or software development methods that involve design practices such as simulation techniques, failure modes effects analysis, statistical techniques to model and analyze process variations etc., as these practices are considered to yield higher confidence in the development. Such development process will be assessed to have higher systemic integrity levels. Further details can be referred to in Section-3 of [17].

**Fig. 1:**
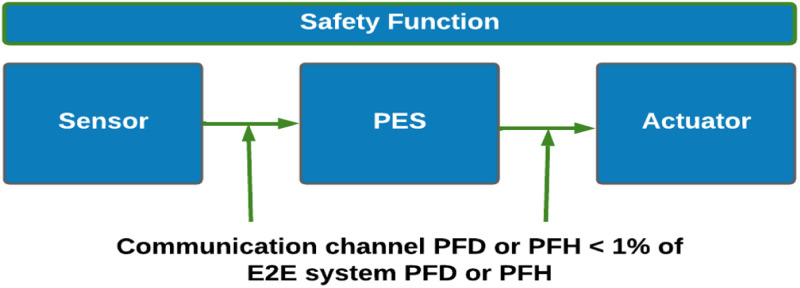
Communication function in safety function.

In the context of QoS for safety systems, ability of WSN depends on the QoS guarantees for latency, error rate, throughput, jitter etc., In applications such as multimedia communication, process control systems, health care systems etc., the vulnerability of WSN to hardware failures, noise interference, cyber-attack could lead to hardware or software failures that might adversely impact the safety functions of the overall systems. To minimize the impact of such failures on safety functions, both hardware and software reliability enhancement techniques are widely used. For example, some hardware reliability enhancement techniques are part stress derating, redundancies /backup architectures, efficient thermal management practices, power saving techniques etc. The software techniques include but are not limited to adopting certain coding guidelines, data transaction protocols with defense mechanisms, software verification & validation methods, failure mode effects testing (FMET), corner testing, fault free testing etc., the above techniques are expected to serve the development of safer systems and help to achieve the QoS guarantees. The QoS objectives of a network can be met by deploying several network management approaches like node clustering, routing, modulation techniques etc. Such approaches are intended to optimize power consumption, minimize latency, data loss, increased throughput etc., In [18] clustering to improve QoS metrics are discussed. The SIL is specified as a probability and depends on the random failure causes. Similarly, QoS metrics of WSN are also probabilistic in nature. The quality of communication links gets affected by various attributes such as channel noise, data dropouts, communication latency, variations in latency referred as jitter, alteration of data sequence, data insertion, masquerade etc. which are referred as the communication threats. Thus, the QoS metrics inherently reflect the ability of an end-to-end system to meet both the performance as well as the safety integrity requirements and can thus be leveraged for safety compliance assessment. To counter the communication threats mentioned above, defense mechanisms are built into the WSN communication systems as brought out in [11]. In communication between source and destination, the communication channel error rate could arise from random hardware failures as well as channel noise. Typical safety communication channels between sensors, controllers and actuators should not contribute more than 1 % to overall system PFD or PFH as indicated in Fig. 1 [19]. The QoS guarantee depends on the application needs. In real time applications such as flight control systems, weapon fire control systems, video streaming for critical procedures, factory automation etc., are characterized by high network demand and hence higher QoS metrics for delay, loss, jitter, and throughput [20] guarantees.

Thus, evaluating the QoS metrics of such systems becomes essential for SIL assurance. The International Society of Automation (ISA) standard practice (SP) work group has published the ISA SP-100 standard for industrial wireless communications. The ISA SP-100 defines 6 classes from Class-0 to Class-5 based on industrial, inter-device wireless communication applications [21]. These classifications are indicated in Table 2. It is intuitive to expect that the class-0 systems need to comply with higher order of SIL as compared to other classes. By implementing one or more remedies such as data packet sequence number, time out with receipt, data consistency check etc., [11] the communication failure modes also referred as communication threats such as repetition, deletion, insertion, masquerade i.e. message mimics etc., could be mitigated. The QoS metrics of a wireless communication system help to assess the degree of guarantee offered as a service against loss of communication, communication delay, throughput and jitter. These could respectively be referred as GoL, GoD, GoT and GoJ.

**Table 2:** Industrial wireless communication application classifications.

Class no.	Applicability	Response time	Application examples
0	Emergency controls	Fast acting systems	• Emergency shutdown systems, • Safety interlocks
1	Closed loop regulatory control	Applications having critical timelines	• Primary controls for pressure, flow motors etc.,
2	Closed loop supervisory control	Applications having longer time constants	• Temperature control systems
3	Open loop control Applications	Human / Operator feedback based (Human in loop) [22]	• Acknowledgement inputs from machine operators • False call acceptance
4	Applications with slow timelines	Monitoring functions with short-term operational consequences	• High & Low Limit alarms • Power ON/OFF indicators • System state indicators
5	Applications without strong timeline criteria	Monitoring functions with no operational consequences	• Downloading • Uploading • Printing

### Industrial application of QOS metrics

Wireless sensor networks are used for several applications including but not limited to personal health monitoring, location detection, movement detection, perimeter monitoring, intrusion detection, military applications etc. [23]. In general, WSN are used in the following broad application areas: rare event detection, periodic data collection, real-time data acquisition, process control, industrial mobile robots, inventory management [21]. The typical QoS metrics used in industrial context, objectives served, and the estimation method are captured in Table 3.

**Table 3:** QOS Metrics used typically in industrial WSN Applications.

QoS Metrics	Objectives	Reference	Estimation method	Typical Application Areas
SDR- Successful Detection Rate	GoL, GoT	[24,25]	Ratio of successful detection of data packets to total data packets detected at the destination node	Streaming Platforms, Video Conferencing, Health care, Environmental Monitoring
FPDR-False Positive Detection Rate	GoT	[24,26,27]	Ratio of number of false detections to number of normal measurements	Intrusion Detection System, Rela time communication, Anomaly detection in cloud computing
TE-Transmission Efficiency	GoT	[28]	Inverse of time taken to complete transmission of all the bytes in a data sequence	Internet protocol, Voice communication, Video streaming
DB-Delay Bound (latency limits)	GoD, GoJ	[[29], [30], [31], [32], [33]]	Time taken for channel access, task scheduling, generation of actuation signal	Cloud computing (data center), edge devices, Video streaming, Real time analytics in autonomous vehicles, Virtual reality
ASE- Area Spectral Efficiency	GoL, GoD	[[34], [35], [36]]	Achieved throughput per square meter (or) bits per symbol per Hz	Cellular networks, Machine-2-Machine communication, Cognitive radio networks
PDR-Packet Delivery Ratio	GoL, GoT	[[37], [38], [39], [40]]	Ratio of packets received at the destination node to packets transmitted from the source node (E2E metric)	Vehicle Ad-hoc Networks (VANETs), Mobile Ad hoc Networks (MANETs), Emergency relief networks and disaster relief
PRR-Packet Reception Ratio	GoL, GoT	[41,42]	Number of packets received at the receiving node to number of packets transmitted from source node	
Data Delivery Reliability	GoL, GoT	[43,44]	Function of E2E delivery rate and Signal to Noise Ratio	Financial Transactions, Health Care Applications, Backup & Recovery. Collaborative remote activities

QoS metrics are critical and are used for ascertaining network performance to comply with the quality & safety targets of the given application. The network management functions include network traffic prioritization, bandwidth allocation for communication, adherence of the network performance per the Service Level agreements, suitability for real time applications etc. [45]. With QoS metrics, the decisions on performance compliance levels of a network are completely data driven which also helps proactive problem solving. For example, building defense measures to protect the network against threats such as Noice interference, data loss, data corruption, masquerade etc., In the following paragraphs the QoS metrics taken to demonstrate the process of safety analysis, its industrial applications, importance and impact of variations of such metrics on the overall applications are discussed.

• Delay Bound (DB):

The delay bound is critical to ensure precise operation of real time functions for example whether a control command is able to reach the actuators or a sensor data is able to reach the controller within the time tolerances specified for the communication. The hard time limits are set for the latency in communication to meet the process time requirements of the safety functions [46] . In case of voice communication, the low latency and jitter to be ensured to avoid voice distortion. Prioritization of destinations, data packets, resource reservation and queuing are some of the techniques adopted to control delay bounds [47]. In machine-to-machine communications, delay bound becomes critical to ensure stability of control loops and avoid processing of outdated data. The maximum delay margin or the maximum tolerable delay beyond which the control loop becomes unstable [48] prevents divergence of control loop

• False Positive Detection Rate (FPDR):

The False Positive Detections in a WSN is a kind of faulty process of measurements in the sensors which could occur due to faulty sensors, noise interference, malicious data etc., [49]. FPDR impacts decision making in WSN as a higher FPDR results in generation of incorrect alerts and warning to the controller or to the operator leading to triggering of corrective actions like back tracking of data, switchover to a degraded performance mode, triggering of backup systems etc. These actions consume resources and results in less efficiency. With deployment of AI for process control, a frequent false alarm could lead to corrupted learning resulting in failure of the system to respond to critical events [50,51]

### Simulation approach

To illustrate SIL assessment of the E2E WSN using QoS metrics, we adopt simulation of QoS metrics by generating random data of False Positive Detection Rate (FPDR) and Delay Bound (DB) probability distributions. The assumed distributions and the settings for simulation cases are indicated in Table 4.

• Simulation of FPDR & DB QoS metrics

**Table 4:** FPDR and Delay Bound (DB) Simulation Cases for a typical SIL-1 system $\left(10^{-02}\leq p<10^{-01}\right)$.

Case No.	FPDR (Binomial Distribution) 50 × 50 Array	DB (Normal Distribution) 50 × 50 Array
1	n = 50, p = 1.0 %, 50 × 50 Array	N (28.125,2.1)
2	n = 50, p = 3.0 %, 50 × 50 Array	N (34.375,2.1)
3	n = 50, p = 6.0 %, 50 × 50 Array	N (40.625,2.1)
4	n = 50, p = 8.0 %, 50 × 50 Array	N (46.875,2.1)
5	n = 50, p = 10.0 %, 50 × 50 Array	N (50,2.1)

The FPDR & DB were simulated as binomially distributed and normally distributed random variables. The FPDR is the ratio of number of false detections to number of normal measurements. From the viewpoint of safety, the FPDR should be lower for a WSN since a false positive detection implies higher probability of error detection even though the actual data packets received are error free. The higher percentage of error detection will demand retransmission of data packets resulting in overall E2E delay in data transfer and processing. Given that the process safety time is the minimum time duration within which mitigation action to be completed by the controller to prevent the hazard from occurring [17], higher delay in data transfer & processing could cause violation of process safety time (PST) resulting in unsafe situations and SIL non-compliance. The major cause for false positive detection is the receiver’s noise figure.

The delay bound (DB) is another QoS metric that has been chosen for the simulation to demonstrate the approach of safety assessment using multi QoS metrics. The delay bound is the time duration between the instant the signal or an event is sensed to the time instant at which the actual action happens. Factors such as channel access delay, receive packet queuing strategy, processing delays etc., contribute to higher latency. The simulation case example illustrated here does not consider impact of factors such as channel noise, transmission protocol, routing algorithms etc. [52]., since absence of such factors do not alter the SIL assessment approach. Moreover, the impact of the later factors could be assessed by introducing these factors as a cascading block in the simulation process. The simulation approach considers SIL-1 targets for demonstrating the proposed approach since these targets are the lowest probability bounds in the industrial safety standard [15] and hence the simulation efforts are minimal to illustrate the approach of SIL assessment.

For each of the simulation test cases, 50 subgroups each subgroup having 50 random samples were generated. Since SIL-1 has the lowest PFD targets, the simulation settings in terms of binomial distribution probability and the mean values of normally distributed DB were chosen within the limits of SIL-1 target range. For example, the p-values (explained in the following paragraph) chosen for simulating the FPDR as a Binomial distribution range from 1.0 % to 10 % which amounts to a success probability of 99 % to 90 % which is within SIL-1 failure probability range. Another example is the false alarm rate of algorithms used for intrusion detection WSN systems and is typically found to be <10 % [53]. As brought out in Table 4, the DB is simulated as a normally distributed variable with its average in the acceptable DB bound of 25msec to 50msec for industrial applications. The standard deviation was chosen as 1/3rd of a quarter range. It is brought out in [54] that the never reducible response time in industrial applications is 100msec and 2–50msec is applicable for the robotic control systems [55,56]. Considering these guidelines, the simulation range for the DB was taken as 25msec to 50msec, which is typically applicable for safety actuation systems.

• The p-value

The output of an experiment is always influenced by noise factors also referred to as random factors. The typical noise factors include but are not limited to machine settings, measurement system errors, environmental factors etc. A common challenge associated with drawing inferences from the experimental results is whether the observed variations in the experimental output are induced by the noise elements, or it is because of the settings of the experimental factors. This ambiguity is resolved by defining a threshold for the probability of obtaining such variations in the results. The probability threshold defines the boundary between common cause induced variations and special cause induced variations and is chosen depending on the required level of confidence in the decision. This probability is estimated by a test statistic considering the distribution and variation in the results to be examined. This process is referred to as hypothesis testing. The hypothesis testing starts by defining 2 hypotheses statements referred to as the ‘Null Hypothesis (Ho)’ and an ‘Alternate Hypothesis (Ha)’. The null hypothesis assumes that the variations observed in the output results are due to the random causes whereas the alternate hypothesis assumes that the variations are not due to random causes rather the variations are due to the special causes, i.e. settings of the experimental factors.

In our case, the objective is to determine whether a specific QoS sample captured belongs to a subgroup of QoS samples captured over a finite time interval or it is a sample so different from the rest of the samples in the subgroup of interest due to special cause induced variations. It may be noted that if the value of QoS sample is significantly different from the average value of the subgroup of QoS samples, then it indicates that the observed QoS sample must not have occurred due to the random causes rather it should have occurred due to some special cause. The special causes could either be from within the network or due to external interference. This is determined by evaluating the Z- statistics of the sample and the corresponding p-value. For 95 % confidence on the decision, if the p-value is less than 0.05, it indicates that the variation of the sample from the subgroup average would have been caused by special causes. On the other hand, if the p-value is greater than 0.05, It indicates that such a variation in the value of QoS sample would have been induced by random causes [[57], [58], [59]]. It can be inferred that when variations in QoS sample values are induced due to special causes, it implies that the defenses in the end-to-end wireless sensor network communication system against the threats such as data drop out, masquerade, data corruption etc., are not adequate to manage the QoS metrics within the safety limits., [11]

• Simulation process flow

**Fig. 2:**
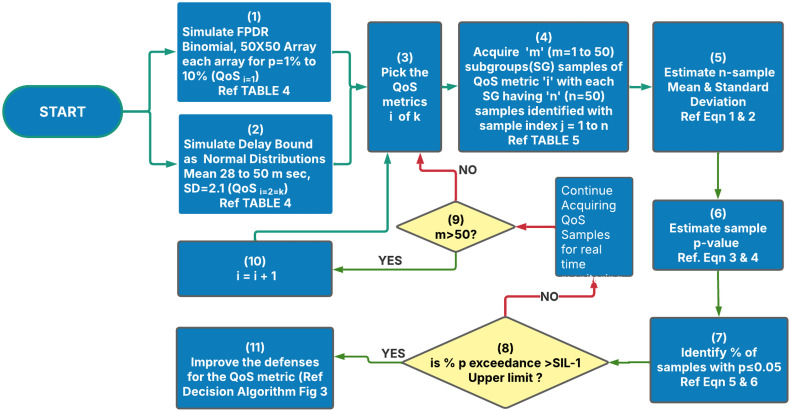
Functional safety assessment process.

Our objective here is to demonstrate the safety assessment methodology when randomly distributed samples of QoS metrics are received in real time in the industrial environment. The processing algorithm to determine SIL compliance is captured in the flow chart shown in Fig. 2

The process steps (1) and (2) indicate the simulation of FPDR and DB QoS metrics. In the actual field environment these blocks will be replaced by data acquisition of respective QoS metrics. From the simulated data of the QoS metric out of ‘k’ metrices (in our case k = 2) that are used to assess the health of an end-to-end (E2E) WSN system, the first one of k QoS metrices is chosen for assessment. In step 3, the samples of the chosen QoS metrices are received for further computations. For illustration purposes, for each of the given simulation test cases shown in Table 4**,** 50 subgroups each having 50 samples of a particular QoS metric amounting to 2500 data points were simulated. The mean and standard deviation (SD) of each of these subgroups were computed. For example, the mean, SD, p-values and % significance are computed as per Eqs. (1) to (5) respectively given below and Table 5 illustrates the subscripts used for sample identification. These computations are represented by process blocks (4)-(7).

(1) $$\begin{array}{c} {\bar{X}}_{m}=\frac{\sum_{j=1}^{j=50}x_{m,\;j}}{50} \end{array}$$

(2) $$\begin{array}{c} \sigma_{m}=\sqrt{\frac{\sum_{j=1}^{j=50}{\left(x_{m,\;j}-{\bar{X}}_{m}\right)}^{2}}{50}} \end{array}$$

(3) $$p_{m\;j}=p\left[Z_{m,j}\right]$$

(4) $$Z_{m,j}=\frac{x_{m,\;j}-{\bar{X}}_{m}}{\sigma_{m}}$$

(5) $$\text{SG}\;\text{Sig}=\frac{\text{Significant}\;\text{number}\;\text{of}\;p\;\text{values}\;\text{in}\;\text{SG}}{\text{No}.\;\text{of}\;\text{Samples}\;\text{in}\;a\;\text{SG}{\;}^{\prime }n^{\prime }}$$

(6) $$\text{Pooled}\;\text{Sig}=\frac{\text{Significant}\;\text{No}.\;\text{of}\;p\;\text{values}\;\text{in}\;\text{all}\;\text{SG}}{\text{No}.\;\text{of}\;\text{Samples}\;\text{in}\;\text{all}\;\text{SG}\;}$$

Where

${\bar{\;X}}_{m}\;\text{is}\;\text{the}\;\text{average}\;\text{of}\;m^{th}\;\text{sub}-\text{group}$
$\sigma_{m}\;\text{is}\;\text{the}\;\text{standard}\;\text{deviation}\;\text{of}\;m^{th}\;\text{sub}-\text{group}$
$x_{m,\;j}\;\text{is}\;\text{the}\;j^{th}\text{sample}\;\text{in}\;m^{th}\;\text{sub}-\text{group}$
$\left[Z_{m,j}\right]is\;the\;Z-value\;of\;the\;j{\;}^{th}sample\;of\;m^{th}\;subgroup$
$p\left[Z_{m,j}\right]is\;the\;probability\;of\;Z-value\;of\;the\;j{\;}^{th}sample\;of\;m^{th}\;subgroup$

**Table 5:** p-value estimation.

Sample ID *j* = *n* → Subgroup ID (m = 1 to 50) ↓	j=1	j=2	…	j=n=50	$\bar{X}$	σ	p-values of samples
m=1	$x_{1,1}$	$x_{1,2}$	…	$x_{1,\;n=50}$	${\bar{X}}_{1}$	$\sigma_{1}$	$p_{1,1}=\frac{x_{1,1}-\bar{X_{1}}}{\sigma_{1}}$ $p_{1,2}=\frac{x_{1,2}-\bar{X_{1}}}{\sigma_{1}}$ … $p_{1,50}=\frac{x_{1,50}-\bar{X_{1}}}{\sigma_{1}}$
m=2	$x_{2,1}$	$x_{2,2}$	…	$x_{2,\;n=50}$	${\bar{X}}_{2}$	$\sigma_{2}$	$p_{2,1}=\frac{x_{2,1}-\bar{X_{2}}}{\sigma_{2}}$ $p_{2,2}=\frac{x_{2,2}-\bar{X_{2}}}{\sigma_{2}}$ … $p_{2,50}=\frac{x_{2,50}-\bar{X_{2}}}{\sigma_{2}}$
…	…	…	…	…	…	…	…
m=50	$x_{50,1}$	$x_{50,2}$	…	$x_{50,\;n=50}$	${\bar{X}}_{50}$	$\sigma_{50}$	$p_{50,1}=\frac{x_{50,1}-\bar{X_{50}}}{\sigma_{50}}$ $p_{50,2}=\frac{x_{50,2}-\bar{X_{50}}}{\sigma_{50}}$ … $p_{50,50}=\frac{x_{50,50}-\bar{X_{50}}}{\sigma_{50}}$

Note: For simulation ‘n’ & m’ are set to n = 50, m = 50.

**Fig. 3:**
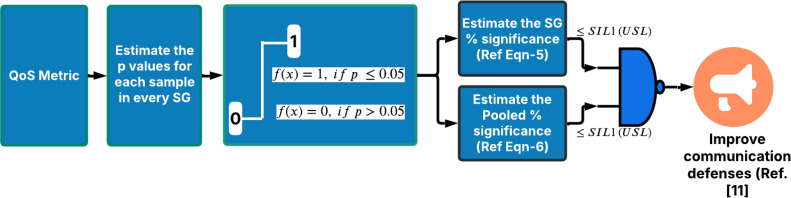
Decision algorithm.

The simulation exercise was carried out using JMP® statistical software [60] and Microsoft® Excel. The FPDR & DB metrics are respectively simulated as binomial and normal distributions. A total of 12,500 random data points were generated for each of these QoS metrics for the study. For the FPDR, each of the data points is generated as an outcome of 50 trials as per the test cases given in Table 4. For each of the test cases, the data is arranged in a 50 × 50 array where each row represents a subgroup starting from 1 to 50 of the specific simulation case, and columns represent the sample IDs 1 to 50. The p-values are then computed for each sample to check if it is significant i.e. if it is <0.05. Once the p-values are computed, the number of p-values that are less than 0.05 were counted. A higher number of significant p-values in a sub-group indicates a higher chance of QoS metrics exceeding the chosen SIL target bounds i.e. SIL-1 in our case. Thus % of p-value exceedance indicates probabilistic health of the QoS metric to meet the safety goals. The lower the %significance better is the health of guaranteeing the QoS metric in the E2E WSN system. These actions are indicated in process blocks (8) and (11) of Fig. 2. Typically, the % significance is chosen as 5 % in our case which can be decided for the application based on the criticality of QoS guarantee for the E2E objectives of WSN system. Thus, compliance of QoS metrics to SIL target values could be assessed by ensuring that the % p-values of each subgroup as well as the % of p values of all the sub-groups pooled are below the upper limits of SIL targets. This generalized decision-making algorithm is shown in Fig. 3. After completing the first cycle of processing ‘m’ subgroup of samples the next QoS metric is chosen for assessment. This process repeats continuously in real time.

### Simulation results

• FPDR results

As explained in Section IV, the FPDR simulation covers the SIL1 probability bounds i.e. 1 % to 10 % to illustrate the process of compliance assessment. This approach of simulating the FPDR over the range is also considered relevant for the likely QoS variations in the application scenario.

From the simulated FPDR 50-subgroup data, p-values were computed, which signifies the probability of observing a particular FPDR when variations are influenced only due to random causes like receiver noise figure, data processing errors etc., Thus, a lower p-value, typically less than 5 %, indicates that the observed variation in FPDR is significant since the associated probability of observing such a FPDR in a sub-group due to random variations is lesser and hence such observations would have been influenced by systematic causes in the WSN system design. Thus, higher proportion of significant p-values in a sub-group indicates poor health of the WSN and calls for immediate improvement in the communication defenses as discussed in [11].

Based on this concept, two metrices as given in Eq. (5) & (6) are evaluated for monitoring the health of the WSN system. The Table 6 provides these metrices i.e. the subgroup and pooled p-values and % significance results. In Table 6, the first column i.e. ‘ % FPDR’ column indicates the binomial distribution probability chosen. The 2nd column, i.e. ‘total number of significant p values from all subgroups’ considers the cumulative count of p-values from all the subgroups. The 3rd column i.e. % significance (pooled)’, is calculated as the ratio of total count of significant p-values (p ≤ 0.05) from all the subgroups to the total simulated values i.e.2500 (50 rows X 50 columns).

**Table 6:** Subgroup significance and Pooled Significance (Typical case).

% FPDR	Total No. of significant p values from all SG	% significance (pooled)	Maximum number of p-values in any SG	% Significance SG Level	SIL-1 Upper Limit
1.00 %	0	0.00 %	0	0.00 %	10 %
3.25 %	0	0.00 %	0	0.00 %	10 %
5.50 %	82	3.28 %	5	10.00 %	10 %
7.75 %	72	2.88 %	5	10.00 %	10 %
10.00 %	96	3.84	4	8.00 %	10 %

**Fig. 4:**
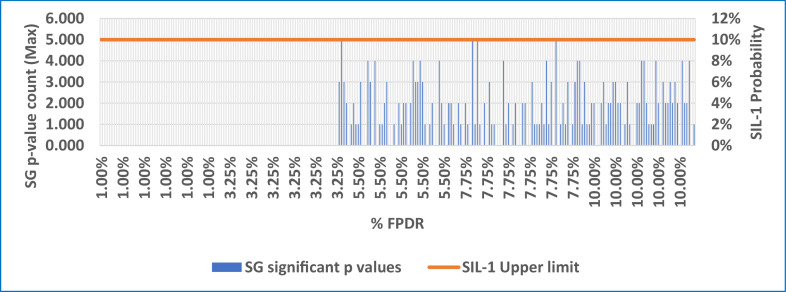
% FPDR Vs SG p- Value count.

**Fig. 5:**
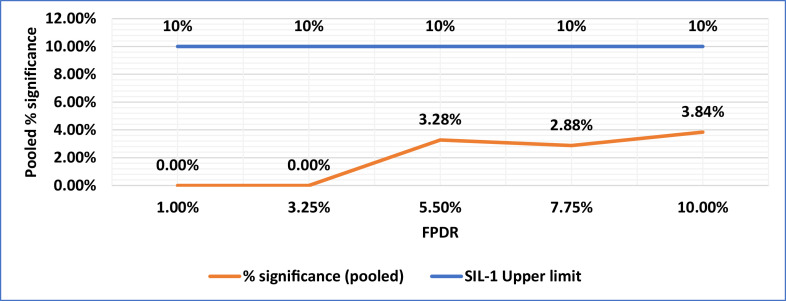
Pooled % Significance Vs % FPDR.

The 4th column captures the maximum number of p-values in the 50 sub-groups. The 5th column, i.e. ‘Significance SG level’ is arrived at as the ratio of maximum number of p-values in any SG to the total elements in a sub-group which is 50. The last column is the upper limit of SIL-1 per IEC 61508 standard. The pooled % significance is found to be lesser than SIL-1 upper limit, indicating that the %FPDR complies with SIL-1 safety goals. The maximum number of p-values count in SG over simulated % FPDR is shown in Fig. 4**.** The SIL-1 upper probability limit is also indicated**.** It can be observed that the % p-value count is less than or equal to the SIL-1 upper limit indicating that the SIL-1 compliance exists up to 10 % of FPDR. This behavior is expected since the max %FPDR chosen for the simulation was only 10 % just equal to SIL-1 upper limit. In the field conditions the variations in %FPDR are very much likely due to factors mentioned in section IV . It can also be observed that at lower %FPDR, the significant p-values do not exist due to lower chances of false positives. The plot of pooled % significance Vs % FPDR is shown in Fig. 5 which indicates that the % significance increases beyond 3.25 % FPDR which is again expected due to increasing probability of false positives. In a typical industrial scenario and for a given protocol of data communication, an FPDR survey measurement will help to determine the safe threshold below which the feasible compliance level of SIL could be achieved without much tuning of the defense mechanisms. In other words, beyond this threshold, defenses against the communication threats need to be enhanced for compliance. In our study, the pooled sample size is chosen as 2500 samples for illustration purposes only. In the application scenario, the required sample size for pooled assessment could be decided considering factors such as application criticality of the WSN communication, data transfer frequency and allowable delay bounds for packet transfer from source to destination nodes.

• Delay Bound simulation

**Fig. 6:**
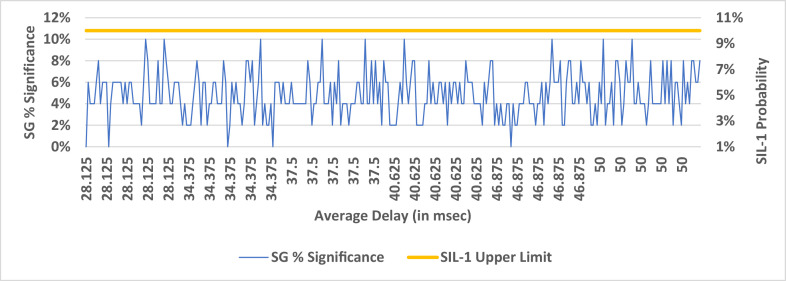
SG % Significance Vs Average Delay in msec.

**Fig. 7:**
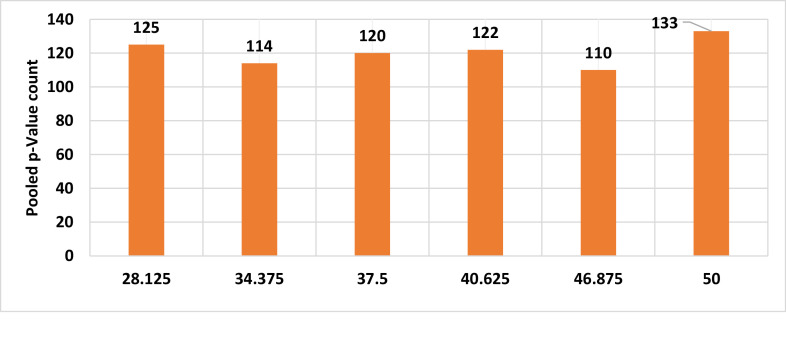
Pooled p-value count Vs Average Delay in msec.

**Fig. 8:**
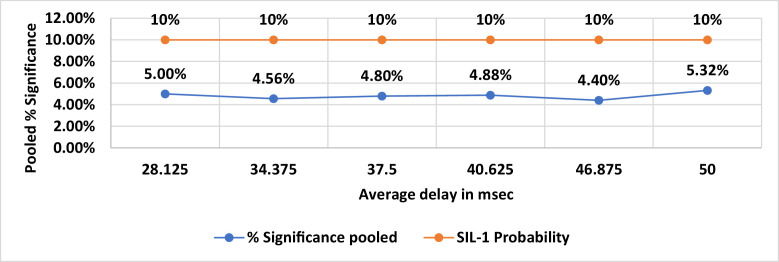
Pooled % Significance.

The delay bound metric assumes higher importance in safety critical communications. As stated in Section IV, latency in data packet reception at the receiver could lead to non-compliance with PST requirements., As explained for the %FPDR simulation, similar plots were constructed for the simulated delay vs p-values and are provided in Fig. 6–Fig. 8. The SG p-values shown in Fig. 6 indicates the maximum number of p- value is found to be 5 which is 10 %, equal to SIL-1 upper limit. 10 % is arrived at as the ratio of maximum number of subgroup p-values to SG size which is 50. From the significant p-value count shown in Fig. 7**,** we find that there is no correlation between the average delay and the pooled p-value count. This could be due to the constant standard deviation chosen for all the normal distributions of delay bounds over the range of 28msec to 50msec. The % significance of pooled p-values are shown in Fig. 8 and is found to be lesser than the upper SIL-1 limits for the chosen standard deviation of delay time. In practice, the jitter (short term variation in delay) could be significantly higher enough to cause exceedance of delay bounds. Under such circumstances, the pooled % p-values will exceed the SIL-1 upper limit thus demanding augmentation of defense mechanisms against the threats. With the chosen standard deviation for delay bounds, these results are found complying with SIL-1 target. In Fig. 7, the pooled p-value counts that are ≤ 0.05 are counted and plotted. The % pooled p-values is arrived at from the ratio of pooled p-value count to the total data points i.e. 2500. For example, at the average delay of 40.625msec, the pooled p-value count is 122 and hence the % pooled p-value ratio is 4.88 % shown in Fig. 8.

### Conclusions and future research opportunities

In this paper we have illustrated a process for functional safety assessment of WSN in industrial applications where multiple QoS metrics are typically expected to be used in the operational scenario. The proposed process has been illustrated by simulating more than one QoS metric viz FPDR and DB. The p-value statistics had been used to assess if the QoS metrics captured over a period in real time comply with the Safety integrity level targets. From the simulated FPDR and DB QoS metrics, compliance with SIL-1 target was assessed by examining the trend plots of these QoS metrics. For the chosen limits of FPDR and DB based on typical industrial scenarios, these metrics were found to be complying with SIL-1 targets. The natural variations in QoS metrics induced by several noise factors in the use scenarios could be differentiated from that of significant degradation in QoS metrics primarily due to inadequate defense mechanisms in the communication, using the p-value statistics discussed in this paper.

The measures such as SG significance and Pooled significance expressions proposed are found to be effective metrics to consolidate the sample p-values for compliance assessment and decision making. With proliferation of WSN in industrial applications, assessing the QoS guarantees for safety management is a compelling need. We are of the view that the illustrated approach may help the safety designers to build analytical models for real-time safety assessment that involves more than one QoS metric to characterize the quality of a WSN. It is suggested that WSN designers can resort to simulation of appropriate distributions of QoS metrics based on limited survey data gathered at the industrial shop floor. The safety assessment concept discussed here is generic and can be adopted for any number of QoS metrics involved in assessing the performance of the WSN. Also, the scope of the illustrated approach could further be expanded to include communication variables such as transaction protocols, channel noise, source and destination node characteristics etc. Finally, we would like to bring out the following summary and future research opportunities in this area of safety assessment of WSN.

• The functional safety assessment of WSN by using the simulated probability distribution data of multiple QoS metrics has been demonstrated which could further be improved by injecting noise-induced variations measured in the shopfloor environment.
• Most of the time typical industrials operational scenarios will involve assessment of multiple QoS metrics for performance assurance of the WSN. Hence the approach adopted to demonstrate safety compliance using 2 QoS metrics is practically relevant. A software tool could be developed for the proposed approach. This tool is expected to help WSN safety engineers to carry out the said simulation for proactive assessment of compliance with SIL and identify the existing threats and required defenses for the communication network.
• The suggested simulation approach to assess SIL safety compliance can also be adopted by network engineers / administrators as part of pre-check or periodical check prior to commissioning the WSN for safety critical tasks.
• The simulation studies discussed in this paper have taken SIL-1 as the target considering the simplicity involved in the simulation to illustrate the concept. As such the same approach could very well be adopted to verify compliance with higher SIL targets i.e. SIL 2–4 as well.
• Use of generative AI solutions to suggest and fine tune the defense mechanisms for safety compliance will be an interesting area to explore further. The results of simulation studies could be used to train the AI model on communication threats and remedial defense mechanisms based on QoS metrics.
• Routing algorithms help to extend the life of nodes and to minimize the latency of data transfer. The simulation approach illustrated in this paper could be linked to routing algorithms to optimize performance for the required level of SIL

In our considered viewpoint, factories of the future will heavily depend on technologies like artificial intelligence, IoT, wireless, digital twins etc., for efficient operation and business success. Hence, deploying the safety assessment methods discussed here will significantly contribute to creating a safer industrial environment.

## Method validation

The safety assessment method discussed in this paper has leveraged simulation of the probability distribution and the process output results are compared against the industrial safety standard i.e. IEC 61508 to determine its compliance.

## Limitations

The methodology illustrated in the manuscript is based on the simulated QoS metrics data generated using random number generation techniques. While care has been taken to simulate the variations typically experienced by the QoS metrics under industrial environmental conditions based on reference publications brought out in this paper, the natural influence due to external noise interference, security attacks, failure condition of network elements, routing consideration induced variabilities etc., will affect the results discussed here. However, the methodology of safety assessment and related algorithms illustrated in this paper will not get affected by said variations and can be deployed to assess SIL of WSN under multi QoS metrics conditions.

## Ethics statements

If your work involved human subjects: None

If your work involved animal experiments: None:

If your work involved data collected from social media platforms: None

## Declaration of competing interest

The authors declare that they have no known competing financial interests or personal relationships that could have appeared to influence the work reported in this paper.

## Data Availability

No data was used for the research described in the article.
